# Real-World Outcomes of Elacestrant in ER+, HER2−, *ESR1*-Mutant Metastatic Breast Cancer

**DOI:** 10.1158/1078-0432.CCR-25-3040

**Published:** 2025-11-13

**Authors:** Hope S. Rugo, Virginia Kaklamani, Heather McArthur, Seth A. Wander, William Gradishar, Reshma Mahtani, Mark Pegram, Maryam Lustberg, Elyse Swallow, Jessica Maitland, Sebastian Kloss, Tomer Wasserman, Sara M. Tolaney

**Affiliations:** 1 City of Hope Comprehensive Cancer Center, Duarte, California.; 2 University of Texas Health Sciences Center, San Antonio, Texas.; 3 UT Southwestern Medical Center, Dallas, Texas.; 4 Massachusetts General Hospital Cancer Center, Boston, Massachusetts.; 5 Robert H. Lurie Comprehensive Cancer Center, Northwestern University, Chicago, Illinois.; 6 Baptist Health Miami Cancer Institute, Miami, Florida.; 7 Stanford Comprehensive Cancer Institute, Palo Alto, California.; 8 Yale Cancer Center, New Haven, Connecticut.; 9 Analysis Group, Boston, Massachusetts.; 10 Analysis Group, Toronto, Canada.; 11 Menarini Group, Florence, Italy.; 12 Menarini Group, New York, New York.; 13 Dana-Farber Cancer Institute, Boston, Massachusetts.

## Abstract

**Purpose:**

The EMERALD trial led to the approval of elacestrant for estrogen receptor (ER)–positive, HER2-negative, estrogen receptor 1 (*ESR1*)–mutated advanced or metastatic breast cancer (mBC) with disease progression following at least one line of endocrine therapy (ET). Subgroup analyses provided evidence suggesting that elacestrant enables ET sequencing in the second line before other targeted combinations, which could delay chemotherapy-based regimens.

**Experimental Design:**

This study used claims data from the Komodo Research Dataset linked with Foundation Medicine Inc. clinical genomic data from patients with estrogen receptor+/HER2− mBC harboring an *ESR1* mutation treated with elacestrant. The primary outcome measure was time to next treatment (TTNT).

**Results:**

Among 306 patients, 93.8% had prior ET ± cyclin-dependent kinase 4/6 inhibitor for ≥12 months, 50.0% had prior chemotherapy, and 72.2% had prior fulvestrant. Median TTNT (mTTNT) was 8.2 months [95% confidence interval (CI), 6.3–13.0] in patients with 1 to 2 prior lines and 7.5 months (95% CI, 7.1–9.9) in those with ≥3 prior lines of ET. In patients with coexisting *ESR1*- and PI3K-pathway–mutated tumors, mTTNT was 6.3 months (95% CI, 4.8–7.9). mTTNT was 7.9 months (95% CI, 7.1–9.8) in all patients and was also sustained in patients with no prior fulvestrant [12.9 months (95% CI, 7.2–not reached)], no prior chemotherapy [8.4 months (95% CI, 7.1–13.3)], visceral metastasis [7.9 months (95% CI, 7.0–9.9)], and liver metastases [7.2 months (95% CI, 6.3–9.0)].

**Conclusions:**

Elacestrant demonstrated a durable benefit in real-world clinical practice, particularly in earlier lines and in patients with prolonged prior ET exposure. Despite coexisting *ESR1* and PI3K pathway mutations, TTNT remained clinically meaningful, reinforcing the role of elacestrant in personalized ET sequencing strategies prior to chemotherapy, antibody–drug conjugates, or targeted combinations.

*See related article by Lloyd et al., p. 169*

**Translational Relevance**

Real-world insights are valuable for affirming the efficacy benefit of elacestrant in estrogen receptor–positive, HER2-negative, estrogen receptor 1 (*ESR1*)–mutant metastatic breast cancer (mBC) in current clinical practice. The primary objective of this clinical and genomic analysis was to describe time to next treatment (TTNT) as a surrogate for PFS in clinically relevant patient subgroups. In a cohort of 306 patients, those with 1 to 2 prior lines of endocrine therapy (ET) achieved an 8.2-month median TTNT with elacestrant (95% confidence interval, 6.3–13.0). In patients with mBC harboring both *ESR1*- and PI3K-pathway–mutated [phosphatidylinositol-3-kinase catalytic subunit alpha (*PIK3CA*), *AKT*, or *PTEN*] tumors, the median TTNT was 6.3 months (95% confidence interval, 4.8–7.9), consistent with clinical trials and independent real-world data. These findings support recommendations to test for *ESR1* mutations in liquid biopsy at each progression and reinforce the role of elacestrant in personalized ET sequencing strategies prior to chemotherapy, antibody–drug conjugates, or targeted combinations.

## Introduction

The current first-line standard-of-care (SOC) treatment for patients with estrogen receptor–positive (ER+), HER2-negative (HER2−), advanced or metastatic breast cancer (mBC) consists of endocrine therapy (ET) combined with a cyclin-dependent kinase 4/6 inhibitor (CDK4/6i; refs. 1–6).

Although this approach improves progression-free survival (PFS) and can increase overall survival, resistance inevitably develops, requiring new treatment strategies. After progression on first-line therapy, the development of resistance affects treatment decisions and becomes more complex due to reduced efficacy and increased toxicity (1–4, 7–13).

Patients with visceral crisis or primary endocrine resistance (progression within 6 months of starting first-line therapy) are not typically candidates for ET-based regimens and benefit more from cytotoxic agents, including antibody–drug conjugates. Upon disease progression after 6 months on first-line ET + CDK4/6i, such patients may become eligible for subsequent ETs, guided by biomarker status (3, 4).

The most frequent mechanisms of intrinsic resistance to ET include activation of the mTOR, PI3K, and AKT signal transduction pathways (14–16). The development of alterations in the estrogen receptor 1 (*ESR1*) gene is a common mechanism of acquired resistance (17–24). *ESR1* mutations develop during ET in the metastatic setting and can be found in up to 40% to 50% of patients at disease progression (18, 25–28). Given the high frequency and availability of effective therapies targeting *ESR1*, current guidelines from the National Comprehensive Cancer Network, American Society of Clinical Oncology, and European Society for Medical Oncology recommend testing for *ESR1* mutations at each stage of metastatic disease progression (1–4). Due to its subclonal nature, the use of archival tissue is not recommended; therefore, ctDNA in liquid biopsy is the preferred testing approach for detecting *ESR1* mutations (29).

Results of the phase III EMERALD trial led to multiple regulatory approvals of elacestrant as the first oral selective ER degrader for the treatment of postmenopausal women or adult men with ER+/HER2− *ESR1*-mutated advanced breast cancer or mBC with disease progression following at least one line of ET (30–32). In the EMERALD trial, single-agent elacestrant was associated with a significant 45% reduction in the risk of progression or death versus SOC ET in patients with ER+, HER2− mBC previously treated with ET + CDK4/6i and who had *ESR1*-mutated tumors [median PFS (mPFS), 3.8 vs. 1.9 months; HR = 0.55; 95% confidence interval (CI), 0.39–0.77; *P* = 0.0005; ref. 32]. Notably, the Kaplan–Meier PFS curves showed an early decline in both arms, suggesting endocrine resistance in some patients (22.6% had received chemotherapy, 23.5% had been exposed to fulvestrant, and 24.3% had primary endocrine resistance in the metastatic setting). In contrast, in patients with more endocrine-sensitive tumors, the clear separation of the survival curves indicates a benefit of elacestrant in ER-driven metastatic disease.

Exploratory subgroup analyses have been performed to evaluate elacestrant outcomes in less endocrine-resistant patient populations (33, 34). In patients with no prior exposure to chemotherapy, an incremental efficacy benefit in mPFS was observed with elacestrant versus SOC (5.3 vs. 1.9 months; HR = 0.52; 95% CI, 0.35–0.75; ref. 33). As EMERALD allowed patients with primary endocrine resistance to be enrolled and considering the majority of the patients are treated in the first line for more than 1 year, outcomes by prior duration of ET + CDK4/6i were analyzed. A clinically meaningful improvement in PFS was observed with elacestrant (*n* = 78) versus SOC (*n* = 81) in patients who had received longer prior ET + CDK4/6i for ≥12 months, leading to 8.6-month mPFS with elacestrant versus 1.9 months with SOC (HR = 0.41; 95% CI, 0.26–0.63; ref. 34). This PFS benefit associated with elacestrant was consistent across clinically relevant subgroups evaluated, irrespective of metastatic site location or number, HER2-low expression, *ESR1-*mutation variant, or coexisting genomic alterations such as *PIK3CA* mutations. Notably, in patients with coexisting *ESR1*- and *PIK3CA*-mutated tumors, mPFS was 5.5 months with elacestrant versus 1.9 months with SOC (HR = 0.42; 95% CI, 0.18–0.94). These results provide early evidence suggesting that elacestrant enables ET sequencing in the second-line setting before other targeted therapies and drug combinations, which may delay chemotherapy-based regimens, including antibody–drug conjugates.

Sufficient time has passed since the approval of elacestrant to further characterize its real-world use in the current treatment landscape. Here, we analyze real-world outcomes with elacestrant in all patients and relevant subgroups, including those with coexisting *ESR1* and PI3K pathway mutations.

## Materials and Methods

### Objectives

This retrospective, observational study aimed to describe the real-world time to next treatment (TTNT) among patients treated with elacestrant in a general population, as well as within clinically relevant subgroups, summarizing different baseline characteristics and genomic data.

### Patients and data source

This analysis utilizes US healthcare insurance claims data from the Komodo Research Dataset (KRD), a Komodo Health data schema designed for real-world evidence and health economic outcomes research studies, linked with Foundation Medicine Inc. clinical genomic (FMICG) data. Patients were selected for inclusion in the analysis if they had been treated for ER+/HER2− *ESR1-*mutated mBC and received elacestrant between January 2023 and February 2025. KRD is a comprehensive and representative data source for identifying treatment patterns and assessing clinical outcomes, healthcare resource utilization, and healthcare costs in the United States. It is sourced directly from more than 150 payers representing more than 165 million lives, as well as data from multiple claims processors, to capture a total of 330 million unique patient journeys. The KRD provides a US census-level representation of all ages, incomes, races, and ethnicities, capturing a large and diverse patient cohort and ensuring socioeconomic diversity by sourcing claims from commercial, Medicare, Medicare Advantage, Medicaid, Managed Medicaid, and other payers, and is generally representative of the demographics and treatment exposures of patients with breast cancer across the United States (Supplementary Table S1). Variables within the KRD include, but are not limited to, inpatient and outpatient diagnoses and procedures, provider information, payer type, mortality data, race, and ethnicity data.

### Clinical outcomes

The index date was defined as the time of the first elacestrant fill. Demographic information at the index date included clinical and genomic characteristics identified during at least a 6-month baseline period before the index date for ET-eligible patients. The first-line setting was defined as the first mBC treatment regimen observed. Subsequent treatment lines were characterized by the initiation of a new drug after at least 30 days from the first fill.

Real-world TTNT was defined as the time from the index date until the initiation of a new line of treatment. The follow-up time for patients without an event was censored at death, the end of clinical activity, the end of data availability, or elacestrant discontinuation, which was defined as a ≥90-day gap from the end of elacestrant days of supply, whichever occurred first. Real-world time to treatment discontinuation (TTD) was defined as the time from index until discontinuation of the index line of therapy. The follow-up time of patients without an event was censored at the earliest occurrence of the end of clinical activity or the end of data availability.

Subgroups of interest were chosen based on clinical relevance and sample size. Patient demographic and clinical characteristics, prior treatment exposure, TTNT, and TTD were summarized for patients in the subgroups.

### Genomics

FMICG mutational data from tissue and plasma samples included *ESR1* mutations (*D538G*, *Y537S*, *Y537N*, *Y537C*, and *E380Q*), *PIK3CA* mutations (*H1047R*, *H1047L*, *H1047Q*, *H1047I*, *H1047Y*, *E545K*, *E545G*, *E545A*, *E545Q*, and *E542K*), *AKT* alterations, and *PTEN* loss of function from patients within the KRD who had received elacestrant at any time and had an eligible line of treatment defined as the initiation of a new drug after at least 30 days from the last fill. Genomic data were sourced from the Foundation Medicine Clinico-Genomic Database (RRID:SCR_017772), which was generated using the FoundationOne assay.

### Statistical analysis

Median TTNT (mTTNT) and median TTD were estimated using the Kaplan–Meier method, and non-adjusted survival curves were generated with 95% CIs. Data analysis was performed using SAS Enterprise Guide 8.3 (SAS Institute Inc.; RRID:SCR_008567) to interface with R Statistical Software (version 4.0.3; R Core Team 2020; RRID:SCR_001905).

### Ethics approval

*KRD* and FMICG are deidentified databases that adhere to Article 164.514(a)-(n)1ii of the US Health Insurance Portability and Accountability Act. Institutional Review Board exemption and patient consent were not required.

## Results

### Demographics

A total of 306 patients with ER+/HER2− mBC and harboring an *ESR1* mutation who initiated elacestrant (between January 2023 and February 2025) were included in this analysis (Supplementary Fig. S1). The median age at the time of elacestrant initiation was 64.4 years, and 98.4% of patients were female (Table 1). Among all patients, 86.9% had visceral metastasis; 45.1% had liver metastasis; 26.8% had lung metastasis; and 19.0% had brain and spinal cord metastasis. Only 12.1% of the patients had bone or lymph-only metastasis. Prior treatments for mBC included ET + CDK4/6i (89.9%), fulvestrant (72.2%), and chemotherapy (50.0%).

**Table 1.** Overall demographics and clinical characteristics.

Parameters	*N* = 306
Median age (years) as of elacestrant initiation	64.4
Female, *n* (%)	301 (98.4)
Race/ethnicity, *n* (%)	​
White (non-Hispanic)	197 (64.4)
Black or African American	34 (11.1)
Hispanic or Latino	23 (7.5)
Asian or Pacific Islander	13 (4.2)
Other	13 (4.2)
Unknown	8 (2.6)
Missing	18 (5.9)
Insurance type, *n* (%)	​
Medicare	183 (59.8)
Commercial	92 (30.1)
Medicaid	29 (9.5)
Missing	2 (0.7)
Metastatic sites, *n* (%)	​
Visceral metastasis	266 (86.9)
Liver	138 (45.1)
Lung	82 (26.8)
Brain and spinal cord	58 (19.0)
Bone/lymph node metastatic site only	37 (12.1)
Bone only	32 (10.5)
Lines of prior ET therapy in mBC, *n* (%)	​
1–2 Lines	128 (41.8)
≥3 Lines	172 (56.2)
Prior mBC treatment use, *n* (%)	​
ET	306 (100)
ET + CDK4/6i	275 (89.9)
ET alone	12 (3.9)
ET ± CDK4/6i ≥ 12 months	287 (93.8)
Fulvestrant	221 (72.2)
Chemotherapy	153 (50.0)
Genomic alterations, *n* (%)	​
*ESR1* mutations	306 (100)
*D538G*	140 (45.8)
*Y537*(S/N/C)	154 (50.3)
Y537S	108 (35.3)
*E380Q*	27 (8.8)
Number of *ESR1* mutation variants	​
1	244 (79.7)
≥2	62 (20.3)
*PIK3CA* mutations	133 (43.5)
*H1047* variants	61 (19.9)
*E545* variants	34 (11.1)
*E542K* variant	19 (6.2)
*PTEN* loss	21 (6.9)
*AKT* alteration	18 (5.9)

### Clinical outcomes

In the overall population (*N* = 306), the median follow-up time was 8.4 months, and the mTTNT with elacestrant was 7.9 months (95% CI, 7.1–9.8). Elacestrant outcomes were evaluated by the number of prior lines of ET received in the metastatic setting. In patients with 1 to 2 prior lines of ET (*n* = 128), mTTNT was 8.2 months (95% CI, 6.3–13.0). If patients were exposed to 1 to 2 prior lines of ET ± CDK4/6i for ≥12 months (*n* = 116), mTTNT was 8.4 months (95% CI, 6.3–14.1). In patients with 1 prior line of ET (*n* = 56), mTTNT was 10.8 months [95% CI, 5.9–not reached (NR)]. In patients who received 1 prior line of an aromatase inhibitor ± CDK4/6i for ≥12 months (83%; *n* = 40), mTTNT was 10.8 months (95% CI, 5.9–NR). In patients who received 1 prior line of fulvestrant ± CDK4/6i for ≥12 months (*n* = 6), mTTNT was 9.9 months (95% CI, 4.6–NR). In patients with two prior lines of ET (*n* = 72), mTTNT was 7.7 months (95% CI, 4.8–13.0). In patients with ≥3 prior lines of ET (*n* = 172), mTTNT was 7.5 months (95% CI, 7.1–9.9).

**Figure 1.**
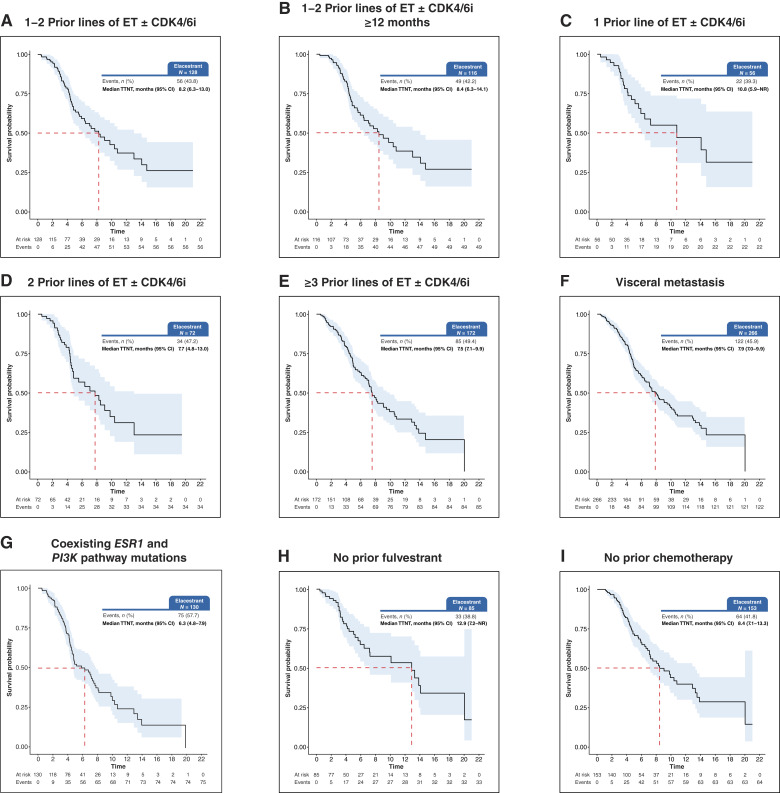
mTTNT benefit in clinical subgroups: (**A**) 1–2 prior lines of ET ± CDK4/6i, (**B**) 1–2 prior lines of ET ± CDK4/6i ≥ 12 months, (**C**) 1 prior line of ET ± CDK4/6i, (**D**) 2 prior lines of ET ± CDK4/6i, (**E**) ≥3 prior lines of ET ± CDK4/6i, (**F**) visceral metastasis, (**G**) coexisting *ESR1* and *PI3K* pathway mutations, (**H**) no prior fulvestrant, and (**I**) no prior chemotherapy.

The mTTNT of patients with visceral metastasis (*n* = 266) was 7.9 months (95% CI, 7.0–9.9). In those with liver metastasis (*n* = 138), the mTTNT was 7.2 months (95% CI, 6.3–9.0). The mTTNT in patients with brain and spinal cord metastasis (*n* = 58) was 9.5 months (95% CI, 5.0–NR). Exposure to prior treatment regimens in the metastatic setting can affect elacestrant performance. For patients with no prior exposure to fulvestrant (*n* = 85), mTTNT was 12.9 months (95% CI, 7.2–NR). The mTTNT of patients with no prior exposure to chemotherapy (*n* = 153) was 8.4 months (95% CI, 7.1–13.3; Table 2; Fig. 1). For patients with prior exposure to fulvestrant (*n* = 221), mTTNT was 7.5 months (95% CI, 6.8–9.0). The mTTNT of patients with prior exposure to chemotherapy (*n* = 153) was 7.4 months (95% CI, 5.6–9.5). TTD in the various clinical subgroups are summarized in Supplementary Table S2 and Supplementary Fig. S2.

**Table 2.** mTTNT benefit in relevant subgroups.

Patient subgroups	*N* (%)	No. of events (%)	mTTNT (95% CI), months
1–2 Prior lines of ET ± CDK4/6i	128 (41.8)	56 (43.8)	**8.2** (6.3, 13.0)
1–2 Prior lines of ET ± CDK4/6i ≥ 12 months	116 (37.9)	49 (42.2)	**8.4** (6.3, 14.1)
1 Prior line of ET ± CDK4/6i	56 (18.3)	22 (39.3)	**10.8** (5.9, NR)
2 Prior lines of ET ± CDK4/6i	72 (23.5)	34 (47.2)	**7.7** (4.8, 13.0)
≥3 Prior lines of ET ± CDK4/6i	172 (56.2)	85 (49.4)	**7.5** (7.1, 9.9)
Visceral metastasis	266 (86.9)	122 (45.9)	**7.9** (7.0, 9.9)
Coexisting *ESR1* and *PI3K*-pathway mutations^a^	130 (42.5)	75 (57.7)	**6.3** (4.8, 7.9)
No prior fulvestrant	85 (27.8)	33 (38.8)	**12.9** (7.2, NR)
No prior chemotherapy	153 (50.0)	64 (41.8)	**8.4** (7.1, 13.3)

a Includes patients with at least one *ESR1* mutation variant (*Y537C*, *Y537N*, *Y537S*, *D538G*, and/or *E380Q*) and at least one *PIK3CA* mutation variant (*H1047*, *E545*, and/or *E542*), *AKT* alteration, or *PTEN* loss of function.

### Genomics analysis

By design, the entire study population had genomic data available for analysis, and all patients (*N* = 306) had tumors with an *ESR1* mutation. The most common *ESR1* mutation allelic variants observed were *D538G* (45.8%), *Y537S* (35.3%), and *Y537N* (14.4%; Table 1). Either *D538G* or any *Y537* variants were found in the majority of the patients. The mTTNT among genomic subgroups was as follows: 7.9 months (95% CI, 6.4–10.6) in patients with *ESR1-*mutation variants *Y537S*, *Y537N*, and/or *Y537C* (*n* = 154) and 8.0 months (95% CI, 7.0–10.5) in patients with the *ESR1-*mutation variant *D538G* (*n* = 140; Supplementary Table S3; Supplementary Fig. S3). In the whole patient population, mTTNT in patients with 1 variant (*n* = 244) was 7.4 months (95% CI, 6.4–9.5), and mTTNT in those with ≥2 variants (*n* = 62) was 10.8 months (95% CI, 7.4–NR; Supplementary Table S3; Supplementary Fig. S3).

mTTNT was 6.3 months (95% CI, 4.8–7.9) in patients (*n* = 130) with coexisting *ESR1*- and PI3K-pathway–mutated (*PIK3CA*, *AKT*, or *PTEN*) tumors (Table 2; Fig. 1). TTD in the various genomic subgroups is summarized in Supplementary Table S4 and Supplementary Fig. S4.

## Discussion

This retrospective, observational study utilizes US healthcare insurance claims data from the KRD and genomics data from FMICG in adults with ER+/HER2− mBC who initiated elacestrant in a real-world setting. The primary objective of this clinical and genomic analysis was to describe TTNT as a surrogate for PFS, offering real-world insights that complement the findings from the phase III EMERALD trial (32, 34).

In US clinical practice, most patients receive ET + CDK4/6i for ≥12 months prior to initiating elacestrant (35). Relative to patients from the EMERALD trial who had *ESR1*-mutated tumors and prior ET + CDK4/6i therapy for at least 12 months, baseline characteristics in this study revealed that most patients had more visceral metastases and received more prior chemotherapy or fulvestrant but had received a similar duration of prior ET + CDK4/6i therapy (Table 3; ref. 32). Despite these differences in clinical characteristics, elacestrant was associated with a longer mTTNT in this analysis compared with the mPFS reported in the EMERALD trial, particularly in those patients who received elacestrant in earlier lines of therapy (Table 4).

**Table 3.** Populations in three analyses.

​	Real-world KRD/FMICG	Real-world GuardantINFORM (36)	Ph III EMERALD subgroup analysis (34)
Baseline characteristics, *n* (%)			
Total patients	306 (100)	756 (100)	78 (100)
Prior CDK4/6i for mBC	275 (90)	624 (83)	78 (100)
Prior ET ± CDK4/6i ≥ 12 months	287 (94)	—	78 (100)
Patients with visceral metastasis	266 (87)	—	58 (74)
Coexisting *ESR1* and *PI3K* pathway mutations^a^^,^^b^	130 (43)	234 (31)	27 (35)
Prior fulvestrant for mBC	221 (72)	402 (53)	13 (17)
Prior chemotherapy for mBC	153 (50)	312 (41)	16 (21)

Abbreviation: Ph, phase.

a Includes patients with *ESR1* mutation variants (*Y537C*, *Y537N*, *Y537S*, *D538G*, and/or E380Q) and *PIK3CA* mutation variants (*H1047*, *E545*, and/or *E542*), *AKT* alteration, or *PTEN* loss of function.

b EMERALD subgroup analysis includes patients with *ESR1*- and *PIK3CA-*mutated tumors.

**Table 4.** Median efficacy outcomes of elacestrant in three analyses.

Patient subgroups	Median efficacy outcomes, months					
	Real-world KRD/FMICG (*N* = 306)		Real-world GuardantINFORM (*N* = 756; ref. 36)		Ph III EMERALD subgroup analysis (*N* = 78; ref. 34)	
	*n*	mTTNT	*n*	mTTNT	*n*	mPFS
1–2 Prior lines of ET ± CDK4/6i	*128*	**8.2**	*—*	*—*	*—*	*—*
1–2 Prior lines of ET ± CDK4/6i ≥ 12 months	*116*	**8.4**	*—*	*—*	*78*	**8.6**
1 Prior line of ET ± CDK4/6i	*56*	**10.8**	*104*	**8.8**	*—*	*—*
2 Prior lines of ET ± CDK4/6i	*72*	**7.7**	*144*	**5.9**	*—*	*—*
≥3 Prior lines of ET ± CDK4/6i	*172*	**7.5**	*492*	**6.4**	*—*	*—*
Visceral metastasis	*266*	**7.9**	*—*	*—*	*—*	*—*
Coexisting *ESR1* and *PI3K-*pathway mutations^a^^,^^b^	*130*	**6.3**	*234*	**5.2**	*27*	**5.5**
All patients with no prior fulvestrant	*85*	**12.9**	*347*	**7.7**	—	—

Abbreviation: Ph, phase.

a Includes patients with *ESR1* mutation variants (*Y537C*, *Y537N*, *Y537S*, *D538G*, and/or *E380Q*) and *PIK3CA* mutation variants (*H1047*, *E545*, and/or *E542*), *AKT* alteration, or *PTEN* loss of function.

b EMERALD subgroup analysis includes patients with *ESR1*- and *PIK3CA-*mutated tumors.

Furthermore, the longer mTTNT in patients without prior exposure to fulvestrant or chemotherapy reflects the benefit of elacestrant in patients with tumors that remain more endocrine-sensitive, retaining ER dependence and less treatment-resistant biology compared with those preexposed to fulvestrant or cytotoxic therapy. This effect was evidenced in the EMERALD trial as well, in which patients with no prior exposure to chemotherapy achieved an mPFS of 5.3 (95% CI, 3.7–9.3) months with elacestrant versus 1.9 (95% CI, 1.9–3.7) months with SOC, HR = 0.53 (95% CI, 0.36–0.80; ref. 33). Patients with no prior exposure to fulvestrant achieved an mPFS of 4.1 (95% CI, 2.2–8.6) months with elacestrant versus 1.9 (95% CI, 1.8–2.1) months with SOC, HR = 0.55 (95% CI, 0.39–0.77). Patients with no prior exposure to fulvestrant achieved an mPFS of 4.1 (95% CI, 2.2–8.6) months with elacestrant versus 1.8 (95% CI, 1.8–2.1) months with fulvestrant, HR = 0.51 (95% CI, 0.35–0.74; data on file).

Findings from our study suggest a consistent benefit associated with elacestrant among relevant subgroups presenting in clinical practice. Specifically, an mTTNT of 8.2 months was observed in patients who had received 1 to 2 lines of prior ET (with 10.8-month mTTNT in those patients with 1 prior line of ET) and 7.5 months in those who received ≥3 lines of ET. An mTTNT of 7.9 months was observed in all patients, with 6.3 months in patients with coexisting *ESR1*- and PI3K-pathway–mutated tumors. An independent, retrospective, observational study was recently conducted using data from the GuardantINFORM database of patients treated with elacestrant who had ER+/HER2− mBC and detected *ESR1* mutations (36). Similar to our analysis, most patients in this study received prior CDK4/6i (83%), prior fulvestrant (53%), and prior chemotherapy (41%; Table 3).

Similar real-world outcomes were observed in this analysis, which also utilized a clinical outcome of real-world TTNT as a surrogate for PFS (36). In the GuardantINFORM study, the mTTNT was 8.8 months in evaluable patients who received elacestrant after one prior line of treatment.

In the KRD/FMICG real-world database, the results showed that a high number of patients (93.8%) have been treated with ET ± CDK4/6i for ≥12 months in real-life settings. The mTTNT of 8.4 months is consistent with the clinically meaningful improvement in mPFS of 8.6 months associated with elacestrant in the EMERALD trial subgroup analysis of patients who received ET + CDK4/6i for ≥12 months (Table 4; ref. 34).

The coexistence of *ESR1* and PI3K-pathway mutations in ER+/HER2– mBC presents a therapeutic challenge, particularly following progression on ET + CDK4/6i. In patients with tumors harboring both mutations, our real-world analysis showed an mTTNT of 6.3 months with elacestrant. This outcome is consistent with the 5.2-month mTTNT reported in the GuardantINFORM database and the 5.5-month mPFS observed in the subgroup of patients with tumors harboring both *ESR1* and *PIK3CA* mutations who had received ≥12 months of prior ET + CDK4/6i therapy in the EMERALD trial (Table 4; refs. 34, 36).

Data from different studies evaluating PI3K/AKT/mTOR pathway inhibitors show similar results in the context of coexisting *ESR1* and PI3K-pathway mutations. In the BYLieve trial, alpelisib combined with ET yielded an mPFS of 4.6 to 5.6 months in patients harboring both *PIK3CA* and *ESR1* mutations previously treated with CDK4/6i (37, 38). Similarly, CAPItello-291 reported a 5.5-month mPFS with capivasertib plus fulvestrant in patients with *PI3K/AKT/PTEN*-altered tumors after CDK4/6i; however, data on coexisting *ESR1* mutations were not reported (13, 39). In the FINER study, ipatasertib plus fulvestrant achieved an mPFS of 5.3 months in all patients and 5.5 months in patients with *PI3K/AKT/PTEN*-altered tumors (40). In patients with *ESR1*-mutated tumors, mPFS was 3.8 months. Notably, toxicity associated with PI3K/AKT/mTOR inhibitors, such as diarrhea, rash, and hyperglycemia, resulted in treatment discontinuation in up to 24% of patients (13, 40, 41).

The role of variant allele frequency (VAF) as a predictor of treatment response in this setting remains an important question. In the EMERALD trial subgroup analysis, elacestrant demonstrated an mPFS of 5.5 months in patients with tumors harboring both *ESR1* and *PIK3CA* mutations. *ESR1*-mutation VAF was lower than *PIK3CA* mutation VAF in 89% of the patients, demonstrating that elacestrant is a viable treatment option in patients with *ESR1*- and *PIK3CA*-mutated tumors, without compromising efficacy despite *ESR1-*mutation VAF being lower than *PIK3CA-*mutation VAF (42). Given that the ER pathway may remain the primary driver of disease and that PI3K/AKT pathway inhibitors are associated with higher toxicities and treatment discontinuation compared with the manageable safety profile of elacestrant, these data support prioritizing elacestrant over initiating PI3K/AKT pathway inhibitors in patients with tumors that have coexisting *ESR1* and PI3K-pathway mutations (13, 34, 41–43).

Although this analysis highlights the activity of elacestrant monotherapy in *ESR1-*mutant, ER+/HER2– mBC, coalterations in the PI3K pathway represent a setting in which rational combinations may provide further options. The ELEVATE study (NCT05563220) is prospectively evaluating elacestrant in combination with alpelisib, capivasertib, and everolimus. Selective PI3K inhibitors in ongoing studies might enable more effective combinations with an improved safety profile.

Observational studies based on real-world data (RWD) provide valuable insights into patient populations encountered in routine clinical practice. When integrated with evidence from randomized controlled trials, they offer a more comprehensive understanding of therapeutic effectiveness and safety in everyday practice. Although RWD analyses can address some of the limitations inherent in clinical trials, they also present distinct methodologic challenges. These include the interpretation of clinical and surrogate outcomes, incomplete or missing data, imprecise or inconsistent definitions of lines of therapy, and issues related to censoring and follow-up duration. Analyses such as TTNT and TTD are commonly used as proxies for PFS. Still, they differ in clinical interpretation and may be influenced by non–disease-related factors. TTNT is an established, robust, and objective real-world endpoint that is used as a surrogate for PFS, as it encompasses the duration of disease control, treatment tolerability, and patient adherence to treatment (44, 45). A systematic review across advanced solid tumor trials found that TTNT correlated strongly with PFS, supporting its value as a surrogate endpoint (46).

### Conclusions

This observational study of real-world use with elacestrant demonstrates outcomes consistent with the EMERALD trial subgroup analysis in patients with prolonged prior exposure to ET + CDK4/6i. Although the EMERALD trial reported an mPFS of 3.8 months in a heavily pretreated ER+/HER2− *ESR1*-mutant mBC population, with 1 to 2 prior lines of ET, the mTTNT of 8.2 months observed in routine clinical practice suggests improved disease control in real-world settings, in line with the subgroup data analysis showing an 8.6-month mPFS, confirming that elacestrant is mainly used in patients with endocrine-sensitive tumors.

In patients with endocrine-sensitive tumors harboring coexisting *ESR1* and *PIK3CA* mutations, the EMERALD subgroup analysis in patients with longer prior ET + CDK4/6i treatment reported an mPFS of 5.5 months with elacestrant. Comparable results were seen in the BYLieve trial, with 5.6 months for alpelisib plus fulvestrant. CAPItello-291 showed 5.5 months for capivasertib plus fulvestrant in *PI3K/AKT/PTEN*-altered tumors and prior exposure to CDK4/6i although data in *ESR1*-mutant mBC are lacking. RWD showed an mTTNT of 6.3 months with elacestrant, supporting similar efficacy to PI3K/AKT inhibitor combinations, but with more manageable tolerability and convenience.

Lastly, the magnitude of benefit seems to be greater in patients with no prior exposure to fulvestrant, with an mTTNT of 12.9 months, and in those with no prior exposure to chemotherapy, with an mTTNT of 8.4 months. A noteworthy observation is the benefit in patients with prior exposure to ≥3 lines, in which elacestrant shows an mTTNT of 7.5 months. Greater clinical benefit with elacestrant was also observed when administered in earlier lines of therapy.

The results of this real-world study exceeded the mPFS of elacestrant in the EMERALD clinical trial and are consistent with the outcomes of the subgroup analysis in patients with endocrine-sensitive tumors, contributing to a growing body of evidence that supports a personalized, biomarker-driven approach to the management of ER+/HER2− mBC.

## Supplementary Material

Supplementary Data 1 Supplemental Figures and Tables

## Data Availability

Data that underlie the results reported in a published article may be requested for products and the relevant indications that have been authorized by the regulatory authorities in Europe/the United States (or, if not, data can be requested for up to 6 years after publication). The Menarini Group will review requests individually to determine whether the requests (i) are legitimate and relevant and meet sound scientific research principles, (ii) are within the scope of the participants’ informed consent, and (iii) are compliant with any applicable laws and regulations and with any contractual relationships that the Menarini Group, its affiliates, and partners have in place with respect to the study and/or the relevant product. Before data are made available, requestors must sign a data-use agreement covering obligations such as compliance with privacy laws, secure handling of data within an approved analysis environment, nonattempted reidentification, and appropriate acknowledgment of the data source. Requests will generally be acknowledged within a reasonable timeframe, and if approved, data access will be provided through a secure platform rather than direct download. Proposals should be directed to medicalinformation@menarinistemline.com.
