# Supplementary material for: Real-World Outcomes of Elacestrant in ER+, HER2−, ESR1-Mutant Metastatic Breast Cancer
Source: Clin Cancer Res. 2025 Nov 13;32(1):179–87. doi: 10.1158/1078-0432.CCR-25-3040 (PMC12770937; doi:10.1158/1078-0432.CCR-25-3040)
Supplement: Supplementary Data 1 — Supplemental Figures and Tables [file ccr-25-3040_supplementary_data_1_suppsd_1.pdf]

Supplemental Figures and Tables

Figure S1: Sample Selection.

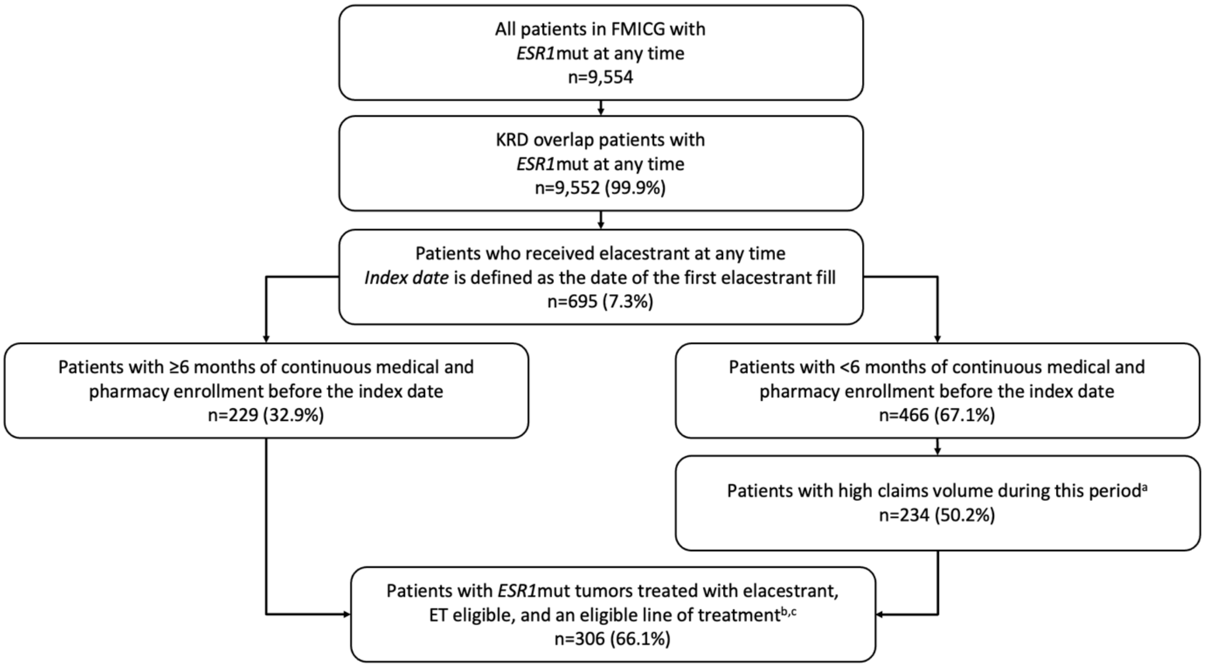

[a] Patients with at least the median number of days with claims (29) among patients with <6 months of continuous medical and pharmacy enrollment before the index date and

[b] An eligible LOT was defined as (1) having at least one claim for elacestrant with non-missing days of supply, and (2) initiating the LOT with elacestrant at least 30 days before the last clinical activity date or end of data. Patients who died within 30 days of the initiation of the LOT with elacestrant were included in the analysis.

[c] The last clinical activity date was defined as the later of the following: the end date of health plan enrollment (if available) or the last day of the month in which the final clinical activity occurred.

*ESR1*mut, estrogen receptor 1 gene mutation; FMICG, Foundation Medicine Inc. Clinical-Genomic; KRD, Komodo Research Dataset.

Figure S2: Median TTD Benefit in Clinical Subgroups. (A) 1-2 prior lines of ET ± CDK4/6i; (B) 1-2 prior lines of ET ± CDK4/6i ≥12 months; (C)1 prior lines of ET ± CDK4/6i; (D) 2 prior lines of ET ± CDK4/6i; (E) ≥3 prior lines of ET ± CDK4/6i; (F) Visceral metastasis; (G) Coexisting *ESR1* and *PI*3K-pathway mutations; (H) No prior fulvestrant; (I) No prior chemotherapy.

Figure S2: Median TTD in Clinical Subgroups

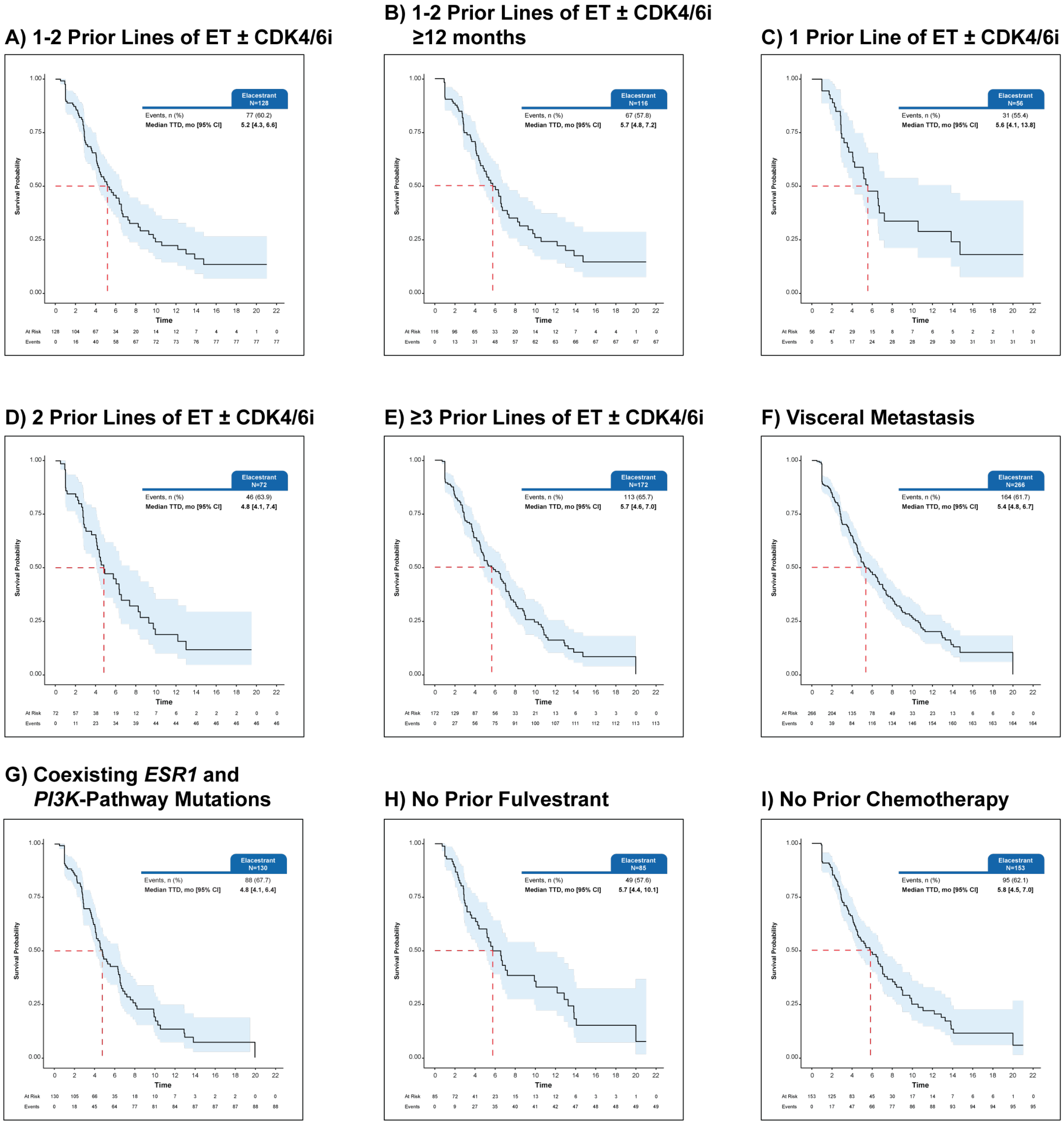

CDK4/6i, cyclin-dependent kinase 4/6 inhibitor; *ESR1*, estrogen receptor 1; ET, endocrine

therapy; PI3K, phosphoinositide 3-kinase; TTD, time-to-treatment-discontinuation.

Figure S3: Median TTNT Benefit in Genomic Subgroups. (A) *ESR1* mutation variants *Y537S* and/or *Y537N* and/or *Y537C*; (B) *ESR1* mutation variant *D538G*; (C) 1 *ESR1* variant; (D) ≥2 *ESR1* variants

Figure S3) Median TTNT in Genomic Subgroups

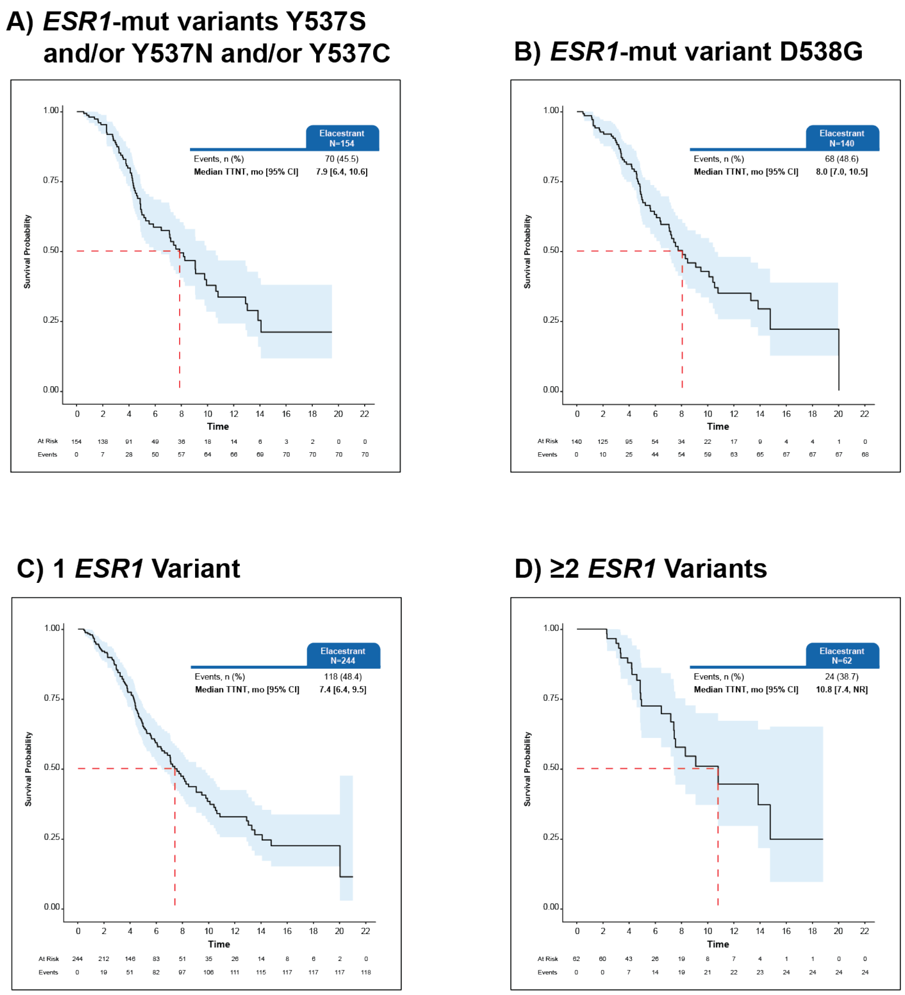

ESR1, estrogen receptor 1; mut, mutation; TTNT, time-to-next-treatment.

Figure S4: Median TTD Benefit in Genomic Subgroups. (A) *ESR1* mutation variants *Y537S* and/or *Y537N* and/or *Y537C*; (B) *ESR1* mutation variant *D538G*; (C) 1 *ESR1* variant; (D) ≥2 *ESR1* variants

Figure S4) Median TTD in Genomic Subgroups

A) *ESR1*-mut variants Y537S and/or Y537N and/or Y537C

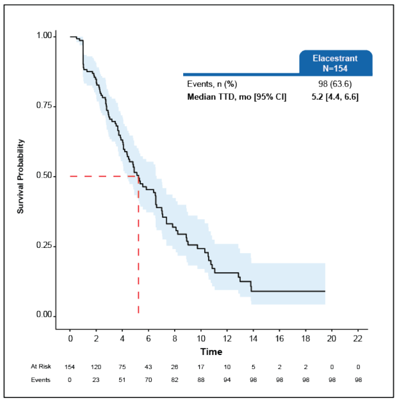

B) *ESR1*-mut variant D538G

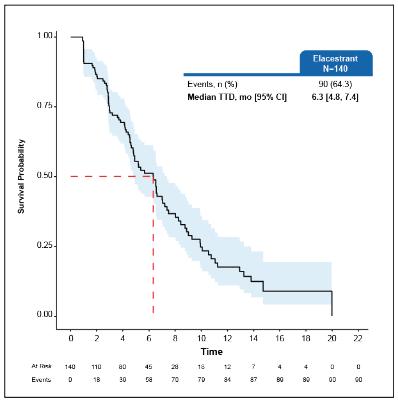

C) 1 *ESR1* Variant

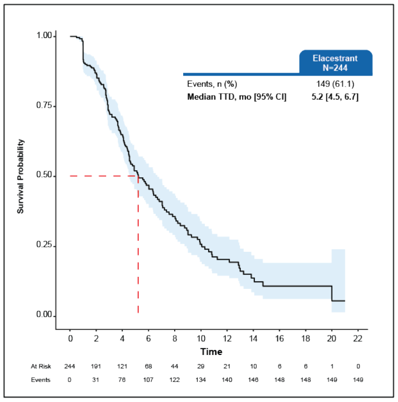

D) >2 *ESR1* Variants

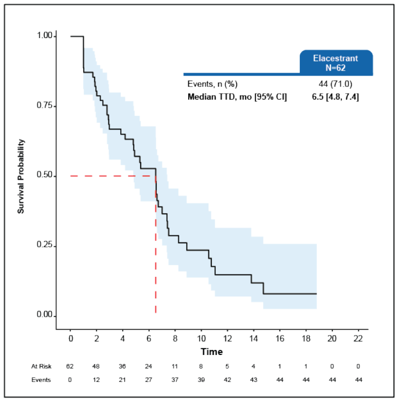

ESR1, estrogen receptor 1; mut, mutation; TTD, time-to-treatment-discontinuation.

Table S1: Representativeness of Study Participants

Cancer type(s) / subtype(s) / stage(s) / condition	Metastatic breast cancer
Special considerations related to:	
Sex and gender	Breast cancer primarily affects women, with about 99% of cases occurring in females.
Age	More than 80% of breast cancers are diagnosed in women aged 50 years or older in the United States. The median age at breast cancer diagnosis is 62 years.
Race or ethnic group	Within the United States, breast cancer occurs most frequently in White and Black women, with incidence rates of 133.7 and 127.8 per 100,000, respectively, while Hispanic and Asian/Pacific Islander women have lower rates at 99.2 and 101.3 per 100,000. Mortality is disproportionately higher among Black women (27.6 per 100,000), which is nearly 40% greater than the rate observed in White women (19.7 per 100,000). Black women are more often diagnosed with de novo metastatic disease compared with White women (8% vs. 5%).
Geography	Breast cancer incidence and mortality differ across countries, impacted by access to early detection strategies and specific risk factors within populations.
Other considerations	Epidemiologic data characterizing metastatic breast cancer in the United States are relatively limited. Statistics from the National Cancer Institute's Surveillance, Epidemiology, and End Results (SEER) program primarily capture patients who present with de novo metastatic disease. However, this represents only a subset of the overall population with advanced breast cancer, as many individuals experience disease recurrence years after treatment for localized disease. Thus, national datasets underestimate the full burden of metastatic breast cancer in the United States.
Overall representativeness of this study	The study population had a median age of 64 years, which is generally aligned with the median age at breast cancer diagnosis in the United States of 62 years. Nearly all participants were female (98%), reflecting the expected sex distribution of breast cancer. and the majority of patients were White followed by Black, Hispanic, and Asian.

Table S2: Median TTD Benefit in Relevant Subgroups.

Patient subgroups	N (%)	No. of events (%)	Median TTD, mo (95% CI)
1-2 prior lines of ET ± CDK4/6i	128 (41.8)	77 (60.2)	**5.2** (4.3, 6.6)
1-2 prior lines of ET ± CDK4/6i ≥12 months	116 (37.9)	67 (57.8)	**5.7** (4.8, 7.2)
1 prior lines of ET ± CDK4/6i	56 (18.3)	31 (55.4)	**5.6** (4.1, 13.8)
2 prior lines of ET ± CDK4/6i	72 (23.5)	46 (63.9)	**4.8** (4.1, 7.4)
≥3 prior lines of ET ± CDK4/6i	172 (56.2)	113 (65.7)	**5.7** (4.6, 7.0)
Visceral metastasis	266 (86.9)	164 (61.7)	**5.4** (4.8, 6.7)
Coexisting *ESR1* and PI3K-pathway mutations[a]	130 (42.5)	88 (67.7)	**4.8** (4.1, 6.4)
No prior fulvestrant	85 (27.8)	49 (57.6)	**5.7** (4.4, 10.1)
No prior chemotherapy	153 (50.0)	95 (62.1)	**5.8** (4.5, 7.0)

[a] Includes patients with at least one *ESR1* mutation variant (*Y537C* and/or *Y537N* and/or *Y537S* and/or *D538G* and/or *E380Q*) and at least one *PIK3CA* mutation variant (*H1047* and/or *E545* and/or *E542*), *AKT* alteration or *PTEN* loss of function.

CDK4/6i, cyclin-dependent kinase 4/6 inhibitor; *ESR1*, estrogen receptor 1; ET, endocrine therapy; mo, months; PI3K, phosphoinositide 3-kinase; TTD, time-to-treatment-discontinuation.

Table S3: Median TTNT Benefit in Genomic Subgroups.

**Genomic subgroups**	**N (%)**	**No. of events (%)**	**Median TTNT, mo (95% CI)**
ESR1 mutation variant *D538G*	140 (45.8)	68 (48.6)	**8.0** (7.0, 10.5)
ESR1 mutation variants *Y537S* and/or *Y537N* and/or *Y537C*	154 (50.3)	70 (45.5)	**7.9** (6.4, 10.6)
1 *ESR1* mutation variant	244 (79.7)	118 (48.4)	**7.4** (6.4, 9.5)
≥2 *ESR1* mutation variants	62 (20.3)	24 (38.7)	**10.8** (7.4, NR)

ESR1, estrogen receptor 1 gene; mo, months; NR, not reached; TTNT, time-to-next-treatment.

Table S4: Median TTD Benefit in Genomic Subgroups.

	**N (%)**	**No. of events (%)**	**Median TTD, mo (95% CI)**
ESR1 mutation variant *D538G*	140 (45.8)	90 (64.3)	**6.3** (4.8, 7.4)
ESR1 mutation variants Y537S and/or *Y537N* and/or *Y537C*	154 (50.3)	98 (63.6)	**5.2** (4.4, 6.6)
1 *ESR1* mutation variant	244 (79.7)	149 (61.1)	**5.2** (4.5, 6.7)
≥2 *ESR1* mutation variants	62 (20.3)	44 (71.0)	**6.5** (4.8, 7.4)

ESR1, estrogen receptor 1 gene; mo, months; TTD, time-to-treatment-discontinuation.
